# Neighborhood Socioeconomic Deprivation is Associated with Worse Cognitive Performance and in vivo Amyloid Accumulation

**DOI:** 10.21203/rs.3.rs-8546338/v1

**Published:** 2026-02-03

**Authors:** Mahsa Mayeli, Riya Saraiya, Adam P. Mecca, David Matuskey

**Affiliations:** Yale University; Yale University; Yale University; Yale University

**Keywords:** Area Deprivation index, MMSE, Positron Emission Tomography, Alzheimer’s disease

## Abstract

**INTRODUCTION:**

Socioeconomic disadvantage (SED) has been associated with poorer brain health, yet its underlying pathology remains incompletely understood. We examined whether neighborhood-level SED, measured using the Area Deprivation Index (ADI), relates to amyloid deposition assessed with amyloid positron emission tomography (PET).

**METHODS:**

Participants (n = 1,110) underwent cognitive assessment using the mini mental state examination (MMSE) and PET scanning with amyloid-specific tracers. Associations between national and state ADI and MMSE and global amyloid burden were evaluated using linear models adjusting for age, sex, and APOE-ε4 carrier status.

**RESULTS:**

In 1,110 participants, higher neighborhood socioeconomic deprivation was associated with lower MMSE scores, with both national and state ADI measures showing significant inverse associations independent of age and sex (all p < 0.001). Higher ADI was significantly associated with greater amyloid burden among cognitively unimpaired participants (β = 0.18, *p* = 0.006, *d* = 0.27), indicating early AD-related pathology.

**DISCUSSION:**

Neighborhood socioeconomic disadvantage is associated with worse cognitive performance and for the first time were shown to be associated with amyloid accumulation during the preclinical phase of AD. These findings underscore the need to consider socioeconomic context in early-stage risk assessment and may help inform targeted prevention strategies aimed at reducing disparities in dementia outcomes.

## Introduction

Socioeconomic disadvantage is consistently linked to poorer health outcomes, including increased rates of cardiovascular disease, diabetes, and cognitive decline [1]–[2]. The Area Deprivation Index (ADI) is a validated, census-based composite measure that captures neighborhood-level socioeconomic disadvantage through indicators such as income, education, employment, and housing quality [3]. Prior studies using ADI and related metrics show that dementias disproportionately affect individuals from economically disadvantaged communities [4] and higher ADI scores have been associated with lower cognitive performance [1, 5–7].

Alzheimer’s dementia (AD), characterized by multidomain cognitive decline [8], currently affects an estimated 6.9 million Americans [8]. Although genetic factors such as the apolipoprotein E ε4 (APOE-ε4) allele are well-established contributors to AD risk and pathology, growing evidence suggests that environmental and socioeconomic exposures may also shape vulnerability to AD [9, 10], potentially through mechanisms involving chronic stress, dietary patterns, toxin exposures, and infectious processes [9–11]. Despite these emerging links, the neurobiological pathways connecting socioeconomic disadvantage to cognitive impairment remain poorly understood.

We examined whether neighborhood-level disadvantage is associated with Alzheimer’s-related pathology by assessing the relationship between ADI and brain amyloid-β (Aβ) burden using positron emission tomography (PET) imaging in cognitively normal (CN) and cognitively impaired (CI) participants from the Alzheimer’s Disease Neuroimaging Initiative (ADNI).

## Methods

### Participants

**Figure 1:**
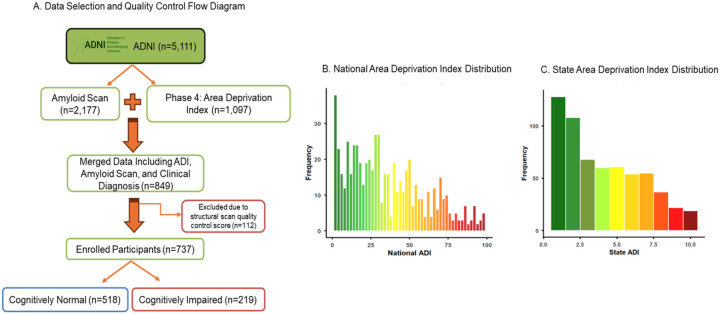
Study population flow and distribution of Area Deprivation Index (ADI) scores.

ADNI participants with complete ADI, amyloid PET data, and clinical diagnosis were included (Fig. 1A). Written informed consent was obtained from all participants, and study procedures were approved by the institutional review boards of all participating institutions. CN individuals had no memory complaints, demonstrated normal memory performance, and showed no cognitive or functional impairment. The CI group consisted of individuals who reported memory concerns, exhibited abnormal memory performance ranging from mild to more pronounced deficits, but retained functional independence [12].

### Cognitive Assessment

Global cognitive function was assessed using the Mini-Mental State Examination (MMSE) [13], a widely used screening tool that evaluates orientation, attention, memory, language, and visuospatial abilities. MMSE scores range from 0 to 30, with higher scores indicating better cognitive performance. MMSE was administered according to standardized procedures by trained study personnel. MMSE scores were treated as a continuous outcome variable in all analyses. Associations between neighborhood socioeconomic deprivation and cognitive performance were examined using multivariable linear regression models, adjusting for age and sex. Participants with missing MMSE or covariate data were excluded from the respective analyses.

### Area Deprivation Index

The ADI data was extracted from ADNI. ADI was calculated using the United States Census indicators of poverty, education, housing, and employment and neighborhood socioeconomic status was ranked by disadvantage at the state and national level. Each census block/neighborhood was split into state deciles and national percentiles, with lower percentile scores indicating less socioeconomic disadvantage.

### APOE Genotyping

*APOE* genotyping was performed using baseline blood samples in accordance with ADNI protocols. Participants were categorized based on APOE ε4 carrier status. Individuals with at least one ε4 allele were classified as APOE ε4 positive.

### Imaging Data Acquisition and Preprocessing

Imaging data processed using the ADNI pipelines (https://adni.loni.usc.edu/data-samples/adni-data/neuroimaging/pet/) were downloaded for these analyses. In brief, two amyloid tracers of [^18^F]florbetapir (FBP) and [^18^F]florbetaben (FBB) were used. PET images were motion corrected before averaging all frames into a single static image. The MRI was segmented and parcellated with Free Surfer v7.1.1 to define a global cortical measure that is made up of frontal, anterior/posterior cingulate, lateral parietal, lateral temporal regions. To generate the standardized uptake value ratios (SUVRs), each amyloid PET scan was co-registered to the corresponding segmented MRI and normalized by the whole cerebellum. SUVR was then calculated for the cortical summary region. Centiloids Click or tap here to enter text.were calculated from cortical summary region SUVRs as described previously [14]. The centiloids scale provides a standardized metric for amyloid PET quantification, where 0 corresponds to amyloid-negative young controls and 100 represents typical amyloid levels in patients with AD. Centiloids calculated based on FBP or FBB PET scans were both included [15]. Additional details can be found at https://adni.loni.usc.edu/data-samples/adni-data/neuroimaging/pet/.

### Statistics Analysis

Data were analyzed in R (v. 4.4.2.). The distribution of all continuous variables was checked for normality using the Shapiro-Wilk test and visual inspection of histograms and Q-Q plots and parametric or non-parametric approaches were selected accordingly. Outlier centiloids (± 3 standard deviations) were removed. Descriptive statistics are reported to summarize the characteristics of the study sample and the distribution of key variables. To examine the predictive value of the ADI in relation to amyloid deposition, linear regression models were employed. Age, sex, and APOE ε4 carrier status were used as covariates (significance: p-value < 0.05).

## Results

In multivariable linear regression models including 1,110 participants and adjusted for age and sex, higher neighborhood socioeconomic deprivation was significantly associated with lower global cognitive performance as measured by the MMSE. Specifically, higher national ADI was associated with lower MMSE scores (β = −0.013, SE = 0.002, p < 0.001), and a similar association was observed for state ADI (β = −0.100, SE = 0.023, p < 0.001). In both models, older age was independently associated with lower MMSE performance (p < 0.001), while sex was significantly associated with MMSE scores (p < 0.001). The overall models explained a modest but significant proportion of variance in MMSE scores (national ADI model: R^2^ = 0.066; state ADI model: R^2^ = 0.060, Table 1).

A total of 737 participants (CN n = 518; CI n = 219) were included (Fig. 1). CN participants were younger than those with CI (*p* = 0.001) and had a lower proportion of males (CN: 189 males, 329 females; CI: 108 males, 111 females; *p* = 0.001). The frequency of APOE ε4 carriers did not differ significantly between groups (CN: 56.7%; CI: 66.7%; *p* = 0.41). Overall amyloid positivity in our study population with a centiloids cutoff of 10 was 32.83%.

**Figure 2:**
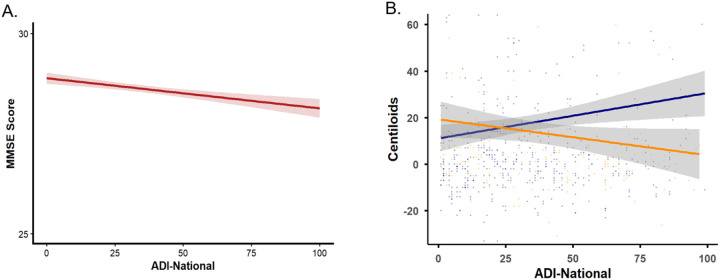
Association between Area Deprivation Index (ADI) Study Measures: A. National ADI and MMSE score; B. National ADI and amyloid burden (Centiloids) in cognitively unimpaired (navy) and cognitively impaired (orange) participants.

Linear regression analyses assessed the relationship between ADI and global amyloid burden (centiloids), controlling for age, sex, and APOE ε polymorphism. In the CN group, both state and national ADI were significantly associated with higher centiloids values (β = 1.38, *p* = 0.01; β = 0.18, *p* = 0.006, respectively). Effect sizes for both state and national ADI were moderate (Cohen’s d = 0.23, 0.27, respectively). In contrast, these associations were not significant in the CI group for state (β = − 1.45, *p* = 0.24) or national (β = − 1.14, *p* = 0.21) ADI (Table 2, Fig. 2). No significant interactions were found between ADI and APOE-ε4 carrier status (p = 0.09).

## Discussion

We investigated the associations between socioeconomic disadvantage and cognition and brain amyloid accumulations. Our findings showed that ADI is significantly associated with worse cognition, and with amyloid burden among CN individuals, even after adjusting for covariates.

Previous studies have addressed the associations between neighborhood deprivation and worse cognition and showed higher odds of cognitive impairment in those participants living in worse neighborhoods [16]. In agreement with previous findings and using the national level ADNI data, we found significant associations between higher state and national ADI and worse MMSE scores.

However, we found no significant associations between ADI and amyloid burden in participants with CI. This could be due to a ceiling effect, meaning that the associations between neighborhood disadvantage and amyloid accumulations cannot be detected as the disease progresses.

These results extend prior work demonstrate that socioeconomic disparities contribute to increased risk of cognitive decline and dementia [2, 4, 6]. Neuropathological studies have reported higher odds of AD pathology with increasing neighborhood disadvantage. Our findings suggest that these associations are detectable through amyloid PET imaging, at least in the preclinical phase of disease. The lack of association in the CI group may reflect that other disease associated processes exert stronger effects when the clinical symptoms have emerged and as disease processes advance, biological and clinical factors may dominate, attenuating the observable influence of socioeconomic context.

In addition, although the APOE-ε4 allele is a well-established genetic risk factor for AD and is associated with greater amyloid accumulation in the brain [17], we did not find any significant interactions between APOE-ε4 carrier status and ADI. Future research should examine potential interactions between polygenic susceptibility and environmental disadvantage and employ longitudinal designs to characterize differential trajectories of amyloid accumulation and the combined effects of gene–environment interactions on disease progression.

Overall, these results reinforce the multifactorial nature of AD risk, highlighting the importance of incorporating social determinants of health into predictive models of neurodegeneration. Future studies integrating environmental, biological, and machine learning approaches may improve our understanding of the pathways through which disadvantage contributes to AD risk and progression.

Our main limitation was the cross-sectional study design, which precludes causal inference. Additionally, larger and more demographically diverse samples are needed to enhance statistical power and explore subgroup-specific effects.

## Figures and Tables

**Table 1:** Associations of National and State Area Deprivation Index (ADI) With MMSE Performance in Models Adjusted for Age and Sex

N = 1,110	Model /Predictor	β	SE	t value	value	R^2^	Adj. R^2^	F statistic
**National ADI**	**ADI (national)**	−0.012	0.002	−5.10	<0.001	0.066	0.064	26.12
	**Age (years)**	−0.054	0.009	−5.88	<0.001			
	**Sex**	0.581	0.136	4.27	<0.001			
**State ADI**	**ADI (state)**	−0.100	0.023	−4.28	<0.001	0.060	0.057	23.45
	**Age (years)**	−0.054	0.009	−5.83	<0.001			
	**Sex**	0.573	0.137	4.19	<0.001			

**Table 2:** Results with ADI as the predictor, centiloids as the outcome and age, gender, and APOE ε polymorphisms as covariates.

Group	Variable	Estimate (β)	SE	t-value	p-value	Cohen’s d
**CN (n = 518)**	Intercept	78.05	14.75	5.29	**0.002**	0.30
	ADI-State	1.38	0.68	2.05	**0.016**	0.23
	Model	75.44	14.80	5.10	**0.004**	0.28
	ADI-National	0.18	0.07	2.62	**0.006**	0.27
**CI (n = 219)**	Intercept	72.52	22.24	3.26	0.740	0.04
	ADI-State	−1.45	0.82	−1.76	0.242	−0.17
	Intercept	68.18	22.54	3.02	0.807	0.03
	ADI-National	−0.14	0.08	−1.69	0.210	−0.18

ADI: Area Deprivation Index; CN: Cognitively Normal; CI: Cognitive Impairment (mild cognitive impairment or mild dementia); SE: Standard Error

## Data Availability

Data were obtained from the ADNI and are available to those researchers with approved data use agreements.

## Highlights

- Higher area deprivation is associated with worse cognition.
- First study linking Area Deprivation Index (ADI) to amyloid PET measures.
- Higher ADI was associated with greater amyloid burden in cognitively unimpaired adults.
- Associations remained significant after adjusting for age, sex, and APOE-ε4.
- Findings suggest socioeconomic context may influence preclinical AD pathology.
